# Complexity of Resting Cortical Activity Predicts Neurophysiological Responses to Theta- Burst Stimulation but Fails to Generalize: A Rigorous Machine-Learning Approach

**DOI:** 10.21203/rs.3.rs-7643216/v1

**Published:** 2025-09-24

**Authors:** Matthew Ning, Haoqi Sun, Brice Passera, Duygu Bagci Das, Brandon Westover, Alvaro Pascual-Leone, Emiliano Santarnecchi, Mouhsin M. Shafi, Recep Ozdemir

**Affiliations:** Beth Israel Deaconess Medical Center; Beth Israel Deaconess Medical Center; Beth Israel Deaconess Medical Center; Beth Israel Deaconess Medical Center; Beth Israel Deaconess Medical Center; Hebrew SeniorLife; Massachusetts General Hospital; Beth Israel Deaconess Medical Center; Beth Israel Deaconess Medical Center

**Keywords:** iTBS, TMS-EEG, MEP, TEP, Machine Learning, Test-Retest iTBS

## Abstract

**Background:**

Substantial variability in individual responses to intermittent theta-burst stimulation (iTBS) limits its clinical efficacy, yet neurophysiological predictors underlying this variability remain unclear. While most machine-learning (ML) studies have focused on modeling behavioral or clinical effects of repetitive transcranial magnetic stimulation (rTMS), the few studies examining neurophysiological outcomes have typically utilized limited feature sets in single-visit settings, which captured only inter-subject variability and most importantly lacked independent validation sets.

**Methods:**

To address these gaps, we first employed statistical and reliability analysis to understand the statistical relationship between resting state EEG and responses to iTBS. Next, we employed supervised machine learning models that integrated baseline resting-state EEG (rsEEG) features and transcranial magnetic stimulation (TMS)-evoked measures, including motor-evoked potentials (MEPs) and TMS-evoked potentials (TEPs), to predict neurophysiological responses to a single iTBS session applied over the primary motor cortex in two independent test-retest studies of healthy adults.

**Results:**

Internal cross-validation within the training cohort yielded promising performance (accuracy: 81%), identifying coarse-grained multiscale distribution entropy of rsEEG as the most robust predictor of local cortical excitability changes indexed by the 100–131ms window of TEPs. However, predictive performance markedly declined upon external validation (accuracy: 69%), reflecting unstable relationships between predictors and outcomes likely driven by substantial intra- and inter-individual variability of iTBS-induced changes in neurophysiological outcomes.

**Conclusions:**

These findings emphasize that while EEG complexity measures can capture baseline brain states relevant for neuromodulation to a certain degree, the inherent instability of single-session iTBS effects significantly constrains model generalizability and underscores the necessity of test-retest paradigm to avoid overly optimistic performance estimates. Future studies with multi-session and individualized stimulation protocols are urgently needed to better characterize neurophysiological mechanisms underlying rTMS effects and ultimately enhance its therapeutic potential.

## Background

Repetitive transcranial magnetic stimulation (rTMS) is a non-invasive neuromodulation technique widely used clinically and experimentally to modify brain excitability, neural plasticity and behavior [1, 2]. Among rTMS protocols, intermittent theta burst stimulation (iTBS) has gained increasing attention due to its relatively shorter duration, lower stimulation intensity, and prolonged effects [3]. Furthermore, it was cleared by FDA to treat depression [4] and has the potential to enhance motor recovery after stroke [5] and improve cognitive performance [6]. Despite these promising outcomes, iTBS has limited efficacy owing to substantial inter- and intra-individual response variability [7–9]. Currently, the mechanisms driving this variability remain poorly understood [10].

Based on the results of invasive repeated electrical stimulation studies in animals, theta-burst stimulation (TBS) protocols are originally considered to modulate behavioral responses by altering neural excitability through Hebbian-like synaptic plasticity mechanisms (cortical excitability hypothesis) [11]. Although initial studies provided partial support for these proposed mechanisms, accumulating evidence shows significant inter- and intra-individual variability in corticospinal and cortical responses [7–9], and many studies failed to demonstrate consistent neurophysiological effects beyond sham controls [12]. One proposed explanation is that neuromodulatory effects depend on the brain’s intrinsic state at the time of stimulation. Optical imaging and electrophysiological recordings in animal models suggest that although evoked responses can be deterministic, variability often arises from the dynamics of ongoing cortical activity [11]. Human TMS-EEG studies similarly show that resting-state EEG (rsEEG) features prior to stimulation correlate with variability in rTMS outcomes, emphasizing the role of intrinsic cortical oscillations in shaping responses to TMS [12–16]. Importantly, recent evidence suggests that specific features extracted from rsEEG can predict individual differences in corticospinal excitability [17] and may reflect both stable and transient neurophysiological characteristics that modulate response to TMS [18].

To date, the majority of studies aiming to characterize inter-individual variability in response to TBS protocol have predominantly focused on predicting behavioral and clinical outcomes using rsEEG metrics. A recent meta-analysis of EEG-based predictive models [19] revealed marked inconsistency across individual studies, reporting highly variable accuracies (approximately 60–90%) and showing that no single EEG-based biomarker has been consistently replicated or validated. In contrast, relatively few studies have attempted to predict neurophysiological outcomes, typically using corticospinal excitability [13–15]. Most existing approaches have relied on linear regression analyses linking baseline motor evoked potential (MEP) amplitudes to post-stimulation changes, which may fail to capture the nonlinear and state-dependent nature of cortical plasticity [16–19]. To the best of our knowledge, only two recent studies have applied machine learning models to baseline neurophysiological features such as MEP amplitudes [20] or resting-state EEG-derived spectral power and complexity measures [21]. Although both studies reported promising internal validation accuracy (76–91%), they lacked external validation using independent cohorts, relied on single-session data capturing only inter-individual variability, and limited their outcomes to corticospinal excitability. These methodological constraints raised concerns about overfitting and generalizability with limited applicability beyond the motor cortex. Moreover, given known limitations of MEPs and the promise of TMS-evoked EEG potentials (TEPs) as direct cortical readouts, it is important to test whether rsEEG complexity features predict iTBS-induced changes in both corticospinal and cortical measures.

Here we evaluate baseline predictors of iTBS-induced neurophysiological change using a test–retest design across two independent cohorts. To address critical knowledge gaps and methodological limitations of previous research, we employed a comprehensive supervised machine-learning approach incorporating multiple neurophysiological metrics including baseline corticospinal and cortical excitability, and rsEEG characteristics. RsEEG features included both conventional frequency-band power metrics and complexity measures to provide an in-depth characterization of baseline cortical activity dynamics. The inclusion of temporal complexity is particularly important, as such nonlinear measures have the potential to capture the brain’s state-dependency on stimulation [22, 23]. Driven by both spectral and nonlinear dynamic components, these measures can detect subtle changes in the EEG signal that conventional spectral properties might miss [24, 25], and have frequently shown equivalent or superior performance in a broad range of EEG applications [24, 26–33]. Unlike previous approaches, our predictive models examined both conventional MEP-based outcomes and TEP-based measures to comprehensively capture iTBS induced changes in cortical neurophysiology. Critically, we trained our predictive models on one cohort and tested the selected model’s performance on the second cohort to test generalization performance and leverage a cross-session paradigm to characterize both inter- and intra-individual variability.

## Methods

### Studies

This study utilized two independent sham-controlled test-retest reliability studies, both collected at Beth Israel Deaconess Medical Center. Cohort 1 was collected from 2018–2021 (unpublished) and Cohort 2 was collected from 2016–2019 [12, 34].

### Participants

For the Cohort 1 Study, data were collected from 28 participants (18 males; mean ± SD age = 39 ± 16 years). For the Cohort 2 Study, data were collected from 24 participants (16 males; mean ± SD age = 30 ± 11 years, range = 18–49). In both studies, all participants were right-handed (assessed by modified Edinburgh handedness inventory) and none had contraindications to TMS or magnetic resonance imaging (MRI), self-reported history of psychiatric or neurological diseases or evidence of drug abuse. In both studies, the participants were not taking any psychoactive medication at the time of measurements. Additionally, caffeine intake, sleep, and menstrual cycle for females were controlled in both cohorts. In accordance with the Declaration of Helsinki, experimental protocols and voluntary participation procedures were explained to all participants before they gave their written informed consent for the study. All questionnaires and procedures were approved by the Institutional Review Board of the Beth Israel Deaconess Medical Center, Boston, MA.

### TBS Procedures

**Figure 1:**
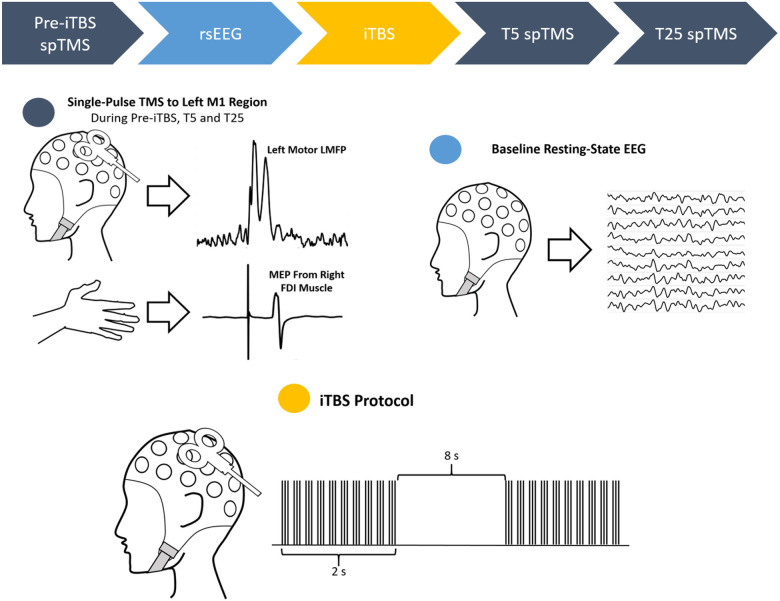
Diagram of iTBS session, applicable to both initial test and retest sessions. Top) Chronological order of sessions with the color of the blocks corresponding to the color of the circles detailing sessions. Navy circle) Three sessions of single pulse TMS were delivered to the left M1 region, one before the iTBS protocol, one 5 minutes after and another 25 minutes after the end of the iTBS protocol. Both EEG and EMG are recorded. Light blue circle) 3 minutes of resting-state EEG with eyes open. Only EEG is recorded. Yellow circle) iTBS protocol consisting a total of 600 bursts spread out in an alternating sequence of trains and silence. Only EEG is recorded.

In both cohorts, each rTMS protocol was administered twice, one for the initial session and another for the retest session. For Cohort 1, five different rTMS protocols (1 Hz, 10 Hz, iTBS, cTBS and sham) were conducted for each participant whereas three rTMS protocols (iTBS, cTBS and sham) were conducted for Cohort 2. Thus, each participant in Cohort 1 underwent a total of 10 TMS sessions and similarly, 6 sessions for Cohort 2. The first block consisted of the initial sessions and the order was randomized for each participant. The second block consisted of retest sessions and the order from the first block was preserved in the second block. To minimize carry-over effect, the visits were spaced at least one week apart for Cohort 1 and at least two days for Cohort 2. Repeated sessions for each rTMS protocol were conducted at least 1 month apart in both Cohorts. Each participant underwent all sessions at roughly the same time of the day to control for possible circadian influences on the neuromodulatory effects of TMS. Only iTBS protocols are used for this report (Fig. 1). All spTMS and rTMS protocols were applied to the left primary motor cortex (M1).

Details of TMS technical specifications and parameters used, determination of motor hotspot, resting motor threshold (RMT) and active motor threshold (AMT), methods used for TEP and MEP recording, preprocessing and analyses are presented in the Supplementary section.

In both studies, single pulses TMS were administered at 120% of the RMT to the motor hotspot. In Study 1, there were three sets of spTMS; a set of 150 pulses delivered before the rTMS protocol (pre-TBS), another set of 150 pulses at 5 minutes after the rTMS protocol (T5), and a third set of 60 pulses at 25 minutes after the rTMS protocol (T25). In Study 2, there’s a total of four sets of spTMS; a set of 120 pulses before the administration of rTMS protocol (pre-TBS), a set of 120 pulses at 5 minutes after the administration of rTMS protocol (T5), a set of 60 pulses at 20 minutes post rTMS protocol (T20) and another set of 60 pulses at 30 minutes post rTMS protocol (T30). In this report, pre-TBS spTMS from both studies were consolidated to make up the Pre-TBS Set, T5 spTMS from both studies were consolidated to make up the T5 Set and T20 spTMS from Study 2 and T25 spTMS from Study 1 were consolidated to make up the T25 Set (Fig. 1, navy circle and arrows).

In both studies, the baseline resting state EEG (eyes-opened) was recorded for three minutes before the application of rTMS protocol but after the baseline single-pulse TMS (Fig. 1, light blue circle and arrow). In both studies, the iTBS protocol was applied to the motor hotspot at 80% of the AMT (Fig. 1, yellow circle and arrow).

### Outcome Measures of iTBS-induced Neuromodulation

As a measure of modulation in corticospinal excitability due to possible neuromodulatory effect of iTBS protocols, for each session, two-tailed two-sample t-test (α ≤ 0.05) was conducted between the MEP peak-to-peak amplitudes of the trials from the Pre-TBS Set and trials from either T5 Set or T25 Set. Two-tailed t-test was used to avoid assumptions about the direction of the modulatory effect. These measures of modulation in corticospinal excitability will be referred throughout here as MEP T5 t-test or MEP T25 t-test. The p-values from these t-tests were used to classify the participant as a responder if the p-value ≤ 0.05 (the MEP peak-to-peak amplitude either significantly increased or decreased after iTBS protocol), else a non-responder.

For the TEPs, local mean field power (LMFP) of different windows was computed as measures of the strength of local cortical activation following TMS pulses. Windows were either determined a priori based on previous literature [35, 36] or systematically tested by looping 30 ms-long window over the 25 to 345 ms period post TMS pulse. Thus, a total of 43 different windows (Supplementary Table S1) were tested in this study. Each window represents a categorization method. The area-under-the-curve (AUC) of the left motor (LM) LMFP of each window was computed using the composite Simpson’s rule from the following EEG channels in the left motor cortex region: C1, C3, C5, FC1, FC3 & FC5 and were averaged across all trials in each block. Next the ratio is taken between the mean AUCs of Post-TBS (T5 or T25 Set) to Pre-TBS Set. The modulation in cortical excitability is classified as facilitation if the LM LMFP ratio for a given window is greater than or equal to 1, and suppression otherwise. These measures of modulation in cortical excitability will be referred throughout here as LM LMFP ratios, with specific windows specified as needed.

### Statistical Tests for Covariate Shift and Label Shift

Supervised algorithms typically depend on the distributions of both the underlying features (\varvec*x*) and response variables (*y*) to be independent and identically distributed across samples. However, strict adherence to assumptions rarely arises in real-world scenarios. For post-hoc analysis, to diagnose poor ML performance, we performed statistical tests for covariate shift and label shift on our datasets. By definition, covariate shift occurs when *p*(\varvec*x*) but not *p*(*y*|\varvec*x*) changed and label shift occurs when *p*(*y*) but not *p*(\varvec*x*|*y*) changed. To test for univariate covariate shift, two-sample Kolmogorov-Smirnov test (α = 0.05) was run for each feature between one sample consisting of the initial test sessions and another sample consisting of the retest sessions. To test for label shift, due to small sample size, two-tailed Fisher’s exact test (α = 0.05) was run instead of chi-squared test between two samples for each categorization method for both corticospinal and cortical excitability.

### Detecting Concept Drift

By definition, concept drift occurs when the posterior distribution *p*(*y*|\varvec*x*) but not *p*(\varvec*x*) changed There’s no formal statistical test for concept drift. Instead, concept drift will be inferred through a combination of aforementioned statistical tests and reliability analysis.

### Reliability Analysis

For post-hoc analysis, for assessment of test-retest reliability, intraclass correlation coefficients (ICCs) based on one-way random effects model (ICC(1,1)) were computed between visits for each band powers, measure of complexity of baseline rsEEG, LM LMFP ratios and MEP T5 and T25 p-values. The p-values of the ICCs are computed using the F-tests. Additionally, Cohen’s kappas were computed between visits using the classifications based on LM LMFP ratios and MEP T5 and T25 t-tests. The p-values of Cohen’s kappa values are computed using the z-tests. Both ICC and Cohen’s kappa values can range from − 1 to 1, endpoints inclusive. Finally, we computed the percentage of individuals with same outcome measures across two sessions (e.g. classified as facilitation using LM LMFP ratio using window 15–45ms in both initial and retest sessions).

### Machine Learning Experiments:

In Experiment 1, called Cross-Session Experiment, initial test sessions from both cohorts make up the training set for the model selection process whereas retest sessions from both cohorts make up the external validation set. Post-hoc analysis will be performed on the results of Experiment 1. In Experiment 2, called Cross-Cohort Experiment, sessions from Cohort 1 make up the training set for the model selection process whereas sessions from Cohort 2 make up the external validation set. Additionally, the visit type (initial test session vs retest session) was encoded and included as an additional feature to capture intersession variability in classifications. Measures of rsEEG spectral powers, temporal complexity, baseline MEPs and LM LMFPs were included as features for MEP and LM LMFP ratio prediction models.

### Baseline rsEEG Band Powers Features

Power spectral densities (PSDs) were estimated from baseline rsEEG using the multitaper method (using Discrete Prolate Spheroidal (Slepian) Sequences as tapers) for each EEG channel and 10 s epochs. Next, the band powers for the four frequency bands are defined as the area under the curve of the PSDs using the following frequency ranges: delta (1–4 Hz), theta (4–8 Hz), alpha (8–12 Hz), beta (12–20 Hz). Finally, the band powers are averaged across epochs for each EEG channel and participant.

### rsEEG Entropy Features

Different measures of complexity (Supplementary Table S2) at fixed temporal scale were extracted from each EEG channel recorded during baseline rsEEG session: approximate entropy [37], sample entropy [38], permutation entropy with embedding dimension 3 [39], distribution entropy [40], incremental entropy [41] and Lempel-Ziv complexity [42]. For multiscale entropy (MSE), complexity indices are computed for permutation entropy of embedding dimension 2 and 3, sample entropy and distribution entropy using coarse-graining [43], time-shifted [44] and composite multiscale procedures [45]. Complexity indices are computed as the AUC of the MSE curve over 20 temporal scales. All complexity measures are computed from a 1-minute recording (first 6 10-s epochs).

### Baseline TMS Features

Four different features based on the MEPs and TEPs from pre-iTBS (baseline) block were computed: the mean peak-to-peak MEP amplitude and its standard deviation, the mean AUC of the LMFP of the LM region using 15 to 80 ms window after the pulses and the mean regression quality score (TEP RQS), a regression-based composite measure of assessing the consistency of individual trials in TEPs [46]. For the MEP Experiments, to capture both local and distal connection, these four features made up the pre-TBS feature set. For the LMFP Ratios Experiments, to capture only the local response, only the mean AUC of the LMFP from the baseline TEPs and the regression quality scores made up the pre-TBS feature set.

### Data Normalization

All features were normalized either using the z-score using the mean and standard deviation of the training set or the distances to the median defined as follows:

$$\tilde{x}=\left|{\text{log}}_{10}x-{\text{log}}_{10}\text{median}(\backslash \text{varvec}X)\right|$$

where **X** represents the training set and will be called Log-Distance transformation.

## ROI

Three different regions of interest are left motor (LM), central and whole-scalp and defined in Supplementary Table S3. Left motor region is our primary ROI as it’s the site of stimulation.

### Classifiers:

Nine different types of classifiers were tested in the model selection step: logistic regression with L2 regularization with inner cross-validation (CV) for hyperparameter tuning; linear discriminant analysis (LDA) with inner CV for hyperparameter tuning; linear discriminant analysis (LDA) using Ledoit-Wolf estimator (LDA LW); LDA using Oracle Shrinkage Approximation estimator (LDA OA); nearest shrunken centroid (NSC) using Manhattan distance metric; NSC using Euclidean distance metric; Gaussian Naïve Bayes (GNB) using empirical priors; GNB using a priori-defined class priors (80 − 20 ratio) and decision tree.

Let TP, TN, FP and FN denote the number of true positives, true negatives, false positives and false negatives. The models were assessed with 7 metrics defined as follows:

$$\text{accuracy}=\frac{TP+TN}{TP+TN+FP+FN}$$

$$\text{sensitivity}=\frac{TP}{TP+FN}$$

$$\text{specificity}=\frac{TN}{FP+TN}$$

$$F_{1}=\frac{2TP}{2TP+FP+FN}$$

$$\text{precision}=\frac{TP}{TP+FP}$$

ROC-AUC, defined as the area under the sensitivity-(1-specificity) curve and PR-AUC, defined as the area under the precision-recall curve. Here, a positive case is defined as p ≤ 0.05 in MEP t-tests or ratio ≥ 1 in the LMFP ratios. While accuracy is used to assess the performance using all predictions from the model, sensitivity and specificity assess the performance of all positive and negative cases correctly identified as positives and negatives, respectively. Precision assesses the percentage of all cases predicted as positive being truly positives. F_1_ score is equivalent to the harmonic mean of precision and sensitivity and is especially useful as a single metric for detecting uneven performance between precision and sensitivity.

10 repetitions of 5-fold stratified cross-validation were used to assess the performance for the model selection. The 95% confidence intervals were estimated using the student’s t-distribution using the sample mean and standard deviation of 50 folds. All models with sensitivity or specificity under 0.60 are excluded for the model selection and model with the highest ROC-AUC was used for model selection during cross-validation. The model selected from cross-validation is then retrained on the entire training set and its performance tested on an external validation set. In this case, bootstrapping was used to compute the 95% confidence intervals, where the external validation set was re-sampled 2000 times, with each re-sampling set stratified to the class proportions of the original sample.

### Feature Grouping and Model Selection:

**Figure 2:**
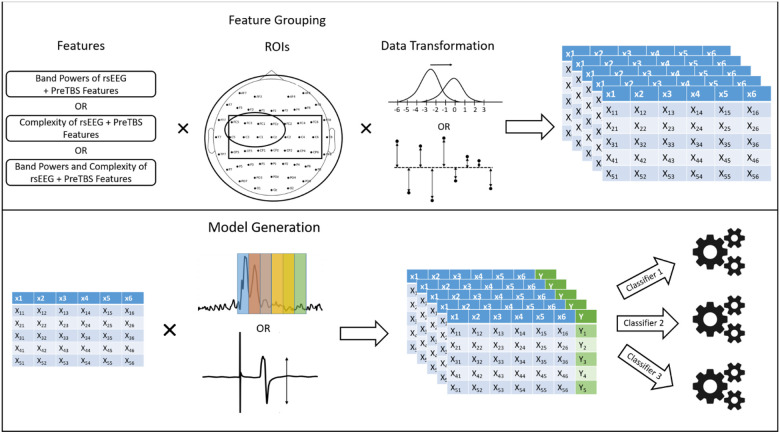
Schematic Diagram of Feature Grouping and Model Generation. In the top panel, feature groups were iteratively generated by generating all combinations of different features (Supplementary Table S2), ROIs (Supplementary Table S3 and data transformations (normalization in top or distance to the median in bottom). Each table represents one feature group. In the bottom panel, models are further iteratively generated by generating all combinations of feature groups, categorization methods (different windows of LMFP in the top (Supplementary Table S1) or t-tests of peak-to-peak MEP amplitudes between post and pre-TBS protocol in the bottom) and classifiers. See the main text for the total number of models tested in MEP and LMFP Ratios Experiments.

Here, we adopt manual feature grouping for feature and model selection. Feature groups are iteratively generated through different combinations of regions of interested (ROIs), data transformations and either powers of frequency bands, measures of complexity or both (Fig. 2). Thus, examples of feature group include 1) z-scores of alpha band powers of rsEEG from the central region, 2) distances to the medians of coarse-graining multiscale sample entropy of rsEEG from the left motor region, and 3) distances to the medians of both beta band powers and approximate entropy of rsEEG from the whole-scalp region. Each feature group represents a set of features to be trained by a classifier. Next, models are iteratively generated by generating all different combinations of feature groups, classifiers and categorization methods of the modulation of corticospinal or cortical excitability.

Thus for the MEP Experiment, a total of 10,152 models (9 types of classifier × 3 ROIs × 2 transformations × 2 post-iTBS MEP blocks (T5 vs T25 blocks) × (4 frequency bands + 18 measures of complexity + (4 × 18)) were trained in cross-validation for the model selection step whereas for LMFP Ratios Experiment, a total of 218,268 models were trained in cross-validation for the model selection step (9 types of classifier × 3 ROIs × 2 transformations × 43 LMFP windows × (4 frequency bands + 18 measures of complexity + (4 × 18)).

### Feature Importance

In LMFP Ratios Experiment, for the decision tree, the feature importance for each feature is estimated using the Gini importance, which is defined as the total reduction of the Gini impurity brought by that feature. This is only available for the decision tree.

## Results

### Analysis Sample

After filtering participants with clean rsEEGs, MEPs and TEPs, the analysis sample consists of 21 participants from Cohort 1, 15 of whom have both initial test and retest sessions, 2 of whom only have the initial test sessions and 4 of whom only have the retest sessions, for a total of 36 sessions, and 19 participants from Cohort 2, 18 of whom have both test and retest sessions and 1 of whom only have the retest session, for a total of 37 sessions. Thus, there is a total of 73 sessions in the analysis sample, with 35 initial test sessions and 38 retest sessions.

### MEP and LMFP Cross-Session Experiments

Briefly, for the MEP Cross-Session Experiment, LDA CV trained on the complexity index of composite multiscale distribution entropy (using Log-Distance transformation) from EEG channels in the left motor region and pre-TBS features, using T25 t-test as the categorization method, has the highest ROC-AUC (mean ± 95% confidence interval: 75.0 ± 6.9, accuracy: 71.3 ± 7.4, sensitivity: 66.0 ± 10.8, specificity: 82.0 ± 11.4, precision-recall AUC: 82.3 ± 4.6, Supplementary Table S4, row a). When tested on the external validation cohort, the performance substantially fell in all metrics (ROC-AUC: 53.6 [36.7, 74.5], accuracy: 52.8 [36.1, 69.4], sensitivity: 47.4 [28.8, 65.9], specificity: 58.8 [35.3, 76.5], precision-recall AUC: 62.7 [51.6, 80.1]) (Supplementary Table S4, row b).

For the LMFP Cross-Session Experiment, when trained on the complexity index of coarse-graining multiscale distribution entropy (using Log-Distance transformation) from the left motor region and pre-TBS features to the TEP ratios using the 105–135 ms window, Logistic Regression with Lasso regularization has the highest cross-validated ROC-AUC (mean ± 95% confidence interval: 83.8 ± 7.4, accuracy: 89.7 ± 4.8, sensitivity: 97.6 ± 2.6, specificity: 70.0 ± 14.1, precision-recall AUC: 90.0 ± 4.4, Supplementary Table S4, row c). Again, when tested on validation cohort, the performance dropped substantially in all metrics (ROC-AUC: 48.6 [39.2, 65.1], accuracy: 60.5 [49.9, 71.1], sensitivity: 90.0 [75.0, 100.0], specificity: 27.8 [16.7, 44.4], precision-recall AUC: 50.8 [48.2, 69.1], Supplementary Table S4, row d), with skew to high sensitivity and low specificity.

The performance drops suggested overfitting but both models from Cross-Session Experiments were relatively small (10 features for MEP Experiment and 8 features for LMFP Experiment) and had regularization. As post-hoc analysis, we performed statistical and reliability analysis, presented in the next two sections.

### Test of Inequality in Distributions

**Figure 3:**
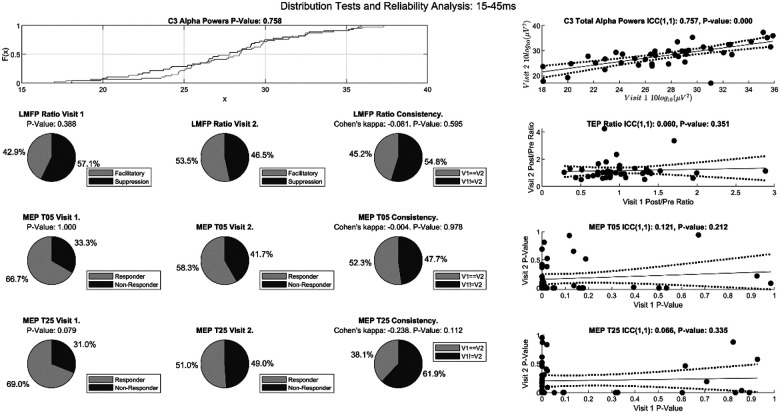
Distribution Tests and Reliability Analysis. A) Empirical distribution functions of the alpha band powers from rsEEG from C3 channel from the initial test sessions (dark gray) and retest sessions (light gray) are shown. Alpha band power from C3 channel is representative of the entire sample. The p value represents the Kolmogorov-Smirnoff test statistic. B) Each pie chart in the left and middle columns displays the class distribution whereas each pie chart in the right column displays the percentage of individuals having consistent outcome measures of iTBS-induced neuromodulation across 2 sessions. The top row represents class distribution determined by the ratio of LMFP of the left motor region for the initial test session (left) and retest session (middle). The 15–45 ms window is picked at random. Similarly, the middle and bottom rows represent the MEP T5 and T25 experiments, respectively. The p values in the left column represent the Fisher’s exact test statistics whereas the p-values in the right column are computed using the z-tests for Cohen’s kappa values. C) Scatter plots with intraclass correlation coefficient (ICC) type (1,1) for the alpha band power from C3 channel (top), LMFP ratio using 15–45 ms window (second from top), p-values of t-tests for MEP T5 block (third from top) and p-values of t-tests for MEP T25 block (bottom). In all 4 cases, the p-values are computed using the F-test. Data interpretation in the main text.

To assess for the presence of univariate covariate shift in rsEEG, univariate two-sample Kolmogorov-Smirnov tests were run between initial test sessions and retest sessions for each EEG channel and feature. Only 2.2% (5/232) of the spectral features have significant differences (without correction) in empirical distribution functions (EDFs) between initial test sessions and retest sessions (see Fig. 3A for a representative channel and band). Similarly, only 3.8% (33/870) of the temporal complexity features of rsEEG have significant differences (without correction) in EDFs between initial test sessions and retest sessions. Both results suggest that their probability distributions remain stable across visits for the majority of the features. To assess for the presence of label shift in the modulations of iTBS protocol, due to small sample size, two-tailed Fisher exact tests (α = 0.05) were ran between initial test sessions and retest sessions. With respect to the LMFP ratios, 4 out of 43 (9.3%) different windows have statistically different class distributions (no correction), and all of them occurred after 200ms post TMS-pulse (see Fig. 3B top row for a representative LMFP window). In both T5 and T25 blocks, the class proportions of MEPs didn’t differ significantly (T5 Block: P-value = 1.000; T25 Block: P-value = 0.079, see Fig. 3B middle row for T5 and bottom row for T25). This suggests that the probability distributions remain stable across visits for the majority of categorization methods.

### Reliability analysis.

The baseline rsEEG band powers generally have high reliability across visits (mean ± SD ICC = 0.83 ± 0.11, no correction, see Fig. 3C top panel for a representative channel and band). Similarly, the temporal complexity of baseline rsEEG have slightly lower but still high reliability across visits (mean ± SD ICC = 0.62 ± 0.24, no correction). Out of 4 different pre-TBS features, only the AUC of the LMFP of the LM region has ICC above 0.3 (ICC = 0.36, P-value = 0.008, RQS: ICC = 0.09, P-value = 0.288, MEP mean amplitude: ICC = 0.06, P-value = 0.338, MEP standard deviation: ICC = 0.03, P-value = 0.417).

The reliability of modulatory effects of iTBS protocol across visits remains low, with the ICC of the MEP T5 −0.01 [−0.30, 0.29] (Fig. 3C, third from top), MEP T25 −0.27 [−0.53, 0.03] (Fig. 3C, bottom panel) and the mean ICC of the LMFP ratios across 43 different windows 0.05 ± 0.11 (Fig. 3C second from top for representative LMFP window). Similarly, Cohen’s kappa results suggested low reliability with the mean Cohen’s kappa of the LMFP ratios across 43 different windows 0.015 ± 0.127 (Fig. 3B, top row). Similarly, Cohen’s kappa for MEP T5 is −0.004 and for MEP T25 is −0.238 (Fig. 3B, middle and bottom rows). The high stability of the probability distributions of spectral powers, different measures of temporal complexity of rsEEG and measures of modulation of corticospinal and cortical excitability and the low reliability of the modulatory effect of iTBS protocol suggested concept drift.

When computing the percentage of individuals with consistent corticospinal and cortical responses across visits for a fixed measure of iTBS-induced modulation, the average percentage across 43 different windows of LMFP ratios is 50.6 ± 6.7% (see Fig. 3D top right for a representative window for LMFP Ratio Experiment). 52.3% and 38.1% of individuals have consistent outcomes across visits for MEP T5 and T25 Experiments, respectively (Fig. 3B, right column).

To address possible concept drift, we change the way dataset is split in order to include both initial test and retest sessions during training. Thus, for Cross-Cohort Experiment, both initial test and retest sessions from Cohort 1 are included in the training set for the model selection step and initial test and retest sessions from Cohort 2 are included in the external validation set for the model validation step. Results are shown in the next two sections.

### MEP Cross-Cohort Experiment

**Figure 4:**
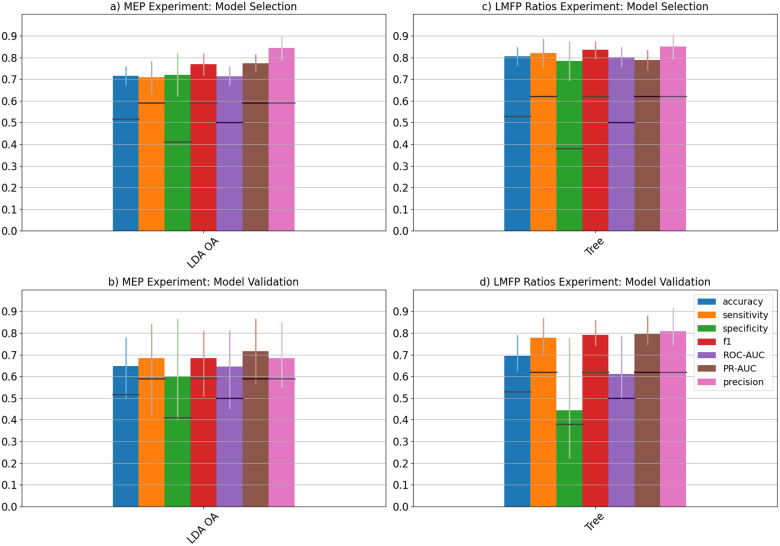
Model Selection and Model Validation Performance of MEP and LMFP Ratios Cross-Cohort Experiments. The top row represents the final models selected during the model selection process (as assessed by cross-validation using ROC-AUC metric), bottom row represents the performance of the final models on validation set during model validation. Left column represents the MEP Experiment whereas the right column represents the LMFP Ratio Experiment. The final model for the MEP Experiment is LDA OA trained on the complexity indices of composite multiscale permutation entropy from all EEG channels whereas the final model for the LMFP Ratios Experiment is decision tree trained on complexity indices of coarse-graining multiscale distribution entropy from EEG channels in the LM region. The vertical error bars represent 95% confidence intervals and the dark horizontal bars within each vertical bars represent theoretical chance levels. 7 different metrics are assessed: accuracy (blue), sensitivity (orange), specificity (green), F_1_-score (red), ROC-AUC (purple), PR-AUC (brown) and precision (pink).

During cross-validation in model selection step, LDA OA trained on the complexity indices of composite multiscale permutation entropy (using normalization) from all EEG channels, using T5 t-test as the categorization method, has the highest ROC-AUC (mean ± 95% confidence interval: 71.4 ± 4.6, accuracy: 71.5 ± 4.5, sensitivity: 70.8 ± 7.6, specificity: 72.0 ± 9.9, precision-recall AUC: 77.4 ± 4.2) (Fig. 4a, Table 1a). When tested on validation cohort, the performance slightly fell in all metrics (ROC-AUC: 64.6 [44.9, 79.6], accuracy: 64.7 [52.9, 76.5], sensitivity: 68.4 [47.4, 84.2], specificity: 60.0 [40.0, 80.0], precision-recall AUC: 71.7 [58.5, 85.4]) (Fig. 4b, Table 1b). The performances are above chance level for all metrics. However, the confidence intervals for all metrics contain chance-levels. C1, P5 and P1 have the largest coefficient magnitudes in the LDA OA model (Supplementary Table S5). The model is relatively large, with 63 features. Coupled it with small drop in performance, the model may be overfitting to the internal validation set.

### LMFP Ratios Cross-Cohort Experiment

The model with the highest cross-validated ROC-AUC is the decision tree trained on the complexity indices of coarse-graining multiscale distribution entropy (using Log-Distance transformation) from the left motor region, with the participants classified using the LMFP ratios computed using the 100–131 ms window (mean ± 95% confidence interval: ROC-AUC: 80.2 ± 4.6, accuracy: 80.6 ± 4.3, sensitivity: 82.0 ± 6.7, specificity: 78.3 ± 9.2, precision-recall AUC: 78.9 ± 4.8) (Fig. 4c, Table 1c). When tested on validation cohort, all performance metrics are above chance-levels (ROC-AUC: 61.1 [49.0, 78.8], accuracy: 69.4 [62.4, 79.2], sensitivity: 77.8 [70.4, 87.1], specificity: 44.4 [22.2, 77.8], precision-recall AUC: 79.5 [74.6, 88.2]) (Fig. 4d, Table 1d). The confidence intervals of accuracy, sensitivity and precision-recall AUC are above chance-levels whereas the confidence intervals of specificity and ROC-AUC contain chance-levels. The model is skewed to high sensitivity and low specificity.

**Figure 5:**
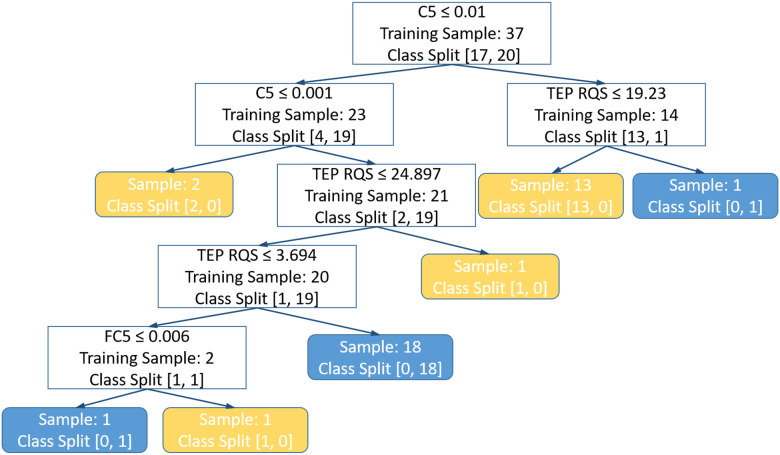
Plot of the final decision tree from LMFP Ratios Cross-Cohort Experiment. If the condition in the top row of the white box is true, the decision path takes the left node, or else, takes the right node. “Training Sample” in the white boxes represents the sample size of the training set before the split. “Class split” represents the class ratio of the training set as follows: [decrease in cortical excitability, increase in cortical excitability]. Yellow box represents a decrease in cortical excitability as assessed by the ratio of the AUC of the 100–131 ms window of the left motor region of LMFP. Similarly, blue box represents an increase in cortical excitability.

Since the decision tree is known to be highly unstable and prone to overfitting, a plot of the final decision tree is shown in Fig. 5, showing that the shortest depth is 2 and that only 3 out of the 9 features are used for classification, with coarse-graining multiscale distribution entropy computed from C5 channel having the highest Gini importance (0.70), followed by the TEP RQS (0.24) and coarse-graining multiscale distribution entropy computed from FC5 channel (0.05) (Supplementary Table S6). Interestingly, visit type isn’t used here. Due to the small size of decision tree, we suspected that concept drift, instead of overfitting, is primarily responsible for the drop in performance in external validation set.

## Discussion

We first trained supervised machine-learning models using baseline features capturing both spontaneous (rsEEG) and stimulus-evoked (MEPs and TEPs) brain dynamics to predict the neurophysiological effects of a single iTBS session applied to the primary motor cortex in two independent cohorts of healthy adults. Internal cross-validation within the first cohort achieved accuracies of 72–81%, comparable to recent reports, and identified coarse-grained multiscale distribution entropy of rsEEG as the strongest predictor of iTBS-induced changes in local cortical excitability. Importantly, the performance of the LMFP Ratios Cross-Session Experiment in the model selection step is comparable or superior to those of previous ML studies [20, 21] that only utilized a single visit. However, when these models were externally validated on the second cohort under near-identical experimental conditions, predictive performance markedly declined, highlighting critical challenges in achieving generalizable models. Although baseline features remained stable, iTBS-induced changes in neurophysiological outcomes exhibited low test–retest reliability with considerable intra- and inter-individual variability, pointing to concept drift, an unstable predictor-outcome mapping, as the primary barrier to model generalization. Future research should systematically examine the mechanisms underlying high response variability using multi-session protocols tailored to individual differences in brain anatomy and physiology to reliably capture neurophysiology of rTMS effects.

Most ML work on rTMS to date has targeted behavioral or clinical endpoints and the few recent studies that model neurophysiological effects of rTMS rely almost exclusively on cortico-spinal responses, lack external validation and are conducted in single-visit setting, omitting intra-individual variability. In order to extend these reports, we modeled both corticospinal and direct cortical responses to iTBS in two independent cohorts. Our results revealed that the temporal complexity of rsEEG was selected over spectral properties of rsEEG in the model selection steps of all 4 experiments, showing that they have higher internal validation performance. This is consistent with previous studies [24, 26–33]. Moreover, in all four experiments, multiscale entropies were selected over single scale entropies. Finally, in both Cross-Session Experiment and Cross-Cohort Experiment, LMFP-based classification of the modulation of cortical excitability outperformed MEP-based classification during cross-validation. In M/EEG studies, different temporal scales of temporal complexity of the brain signal were shown to be linked to different scales of cortical processing, with the structure of variability at short time scales, or high frequencies, linked to local neural population processing, and the variability at longer time scales, or lower frequencies, linked to large-scale network processing [47–49]. Furthermore, we found that the best ML performance was for the LMFP ratios based on the 100–131ms time window following TMS pulses (Accuracy: 0.81), suggesting that N100 peak responses may play a role in the modulatory effects of iTBS. The N100 is one of the most robust and widely studied TEP components, consistently elicited from multiple sites of stimulation including motor, prefrontal, parietal, or cerebellar stimulation sites and is tightly linked to GABAergic mediated cortical inhibition [50]. While iTBS protocol to M1 region is well studied [51], reports on the specific effect on N100 are very limited and inconsistent. For instance, one study reported a non-reproducible increase of N100 amplitude [12], while another one found a significant reduction [52]. These heterogeneous results suggest that a single session of iTBS protocol to M1 cortex does not have a reproducible effect on N100 peak and likely explain the performance drop we observed during external validation.

The marked discrepancy in predictive performance between internal cross-validation and external validation highlights the challenges of developing generalizable prediction models for iTBS-induced neurophysiological effects. While overfitting is a common reason for the drop in performance, we do not suspect substantial overfitting here due to the relatively small size of the models. To better understand the potential source of performance drop in the validation, we assessed covariate shift by evaluating the stability of baseline features (rsEEG, MEPs, and TEPs) across visits and label shift by examining potential changes in outcome class distributions. First, using univariate Kolmogorov-Smirnov tests, we found that the distributions of spectral powers and temporal complexity of baseline rsEEG generally stay stable across initial and retest samples, ruling out univariate covariate shift. This is consistent with previous studies [53, 54]. Using Fisher’s Exact Test, few measures of the modulation of cortical excitability were found to have significantly different class proportions between initial and retest samples and all of them occur after 200 ms after the TMS pulse. This ruled out label shifts for the LMFP periods before 200 ms post-TMS-pulse and is consistent with previous study [55]. Importantly, we found low reliability in the modulatory effect of iTBS protocol, as assessed by the intraclass correlation coefficients and Cohen’s kappa values. These findings collectively point toward concept drift, an unstable or inconsistent relationship between predictive features and outcomes, as the primary driver of poor generalization. Although we did not directly quantify temporal shifts in feature-outcome relationships, the stability of baseline predictors and the group-level consistency of outcome classes strongly imply that individual-level variability in response to iTBS underlies the observed drift.

Concept drift could arise for a number of reasons. For instance, our chosen outcome measures (MEPs and TEPs) of cortical excitability may not fully capture the neurophysiological effects of iTBS, suggesting a potential mismatch between measured outcomes and actual neuromodulatory processes. Therefore, the observed concept drift may partially reflect limitations of our current neurophysiological measures in accurately and consistently indexing iTBS effects across individuals and sessions. The cortical-excitability hypothesis was formulated largely on the basis of early human motor-cortex studies in which single-session rTMS produced group-level changes in MEP amplitudes with high-frequency or patterned rTMS (e.g., iTBS) increasing corticospinal excitability [3] while low-frequency protocols produced the opposite effect [56]. However, these early seminal reports were typically under-powered, lacked robust sham controls, and were not replicated across repeat sessions. Indeed, more recent studies with larger sample sizes and repeat session sham-controlled designs showed that single session of rTMS protocols, including iTBS, do not consistently modulate canonical measures of cortical excitability beyond robust sham protocols in healthy participants and often show poor test-retest reproducibility across identical visits [12]. These reports raises the possibility that MEP and TEP measures may be *insufficient* physiological read-outs for capturing the main biological actions of rTMS. Recent mechanistic reviews indicates that rTMS induces a far richer array of neural changes than simple shifts in excitability. These effects include the widespread modulation of neurotransmitter systems like dopamine [57] and serotonin [58, 59], the triggering of activity-dependent gene expression [60, 61] and epigenetic remodeling [62, 63], the release of neurotrophic factors like BDNF [64, 65], and the engagement of broader neuro-endocrine [66–68] and glial pathways [69].

Several alternative explanations should also be considered. One possible explanation is that, even if the cortical-excitability hypothesis remains partially valid, a single session of iTBS may be insufficient to reliably induce measurable changes in MEP or TEP indices. In clinical practice, rTMS treatments typically involve multiple daily sessions over several weeks, whereas most experimental studies evaluate neuromodulatory effects using a single pre-post stimulation design and track responses over 60–90 minutes. Animal research has demonstrated that different stimulation doses engage distinct neural mechanisms [70]. While a single high-frequency rTMS session primarily affects transient membrane potentials and ionic currents, repeated daily sessions may drive alterations in neurotransmitter receptor levels [71–74] and sustained neurotrophic signaling, such as BDNF elevation [64, 65], leading to stable and durable network reorganization. Consequently, the short-lived, activity-dependent plasticity after single-session rTMS protocols likely exhibits considerable variability both within and between individuals, whereas repeated sessions may generate cumulative and more reliable neurophysiological changes. Interestingly, one study showed that multiple blocks of iTBS in a day does not improve reliability [75]. However, that study doesn’t use the same volume of essions spread over multiple days as the clinical studies do. Another potential explanation might be the sensitivity of our features to detect iTBS induced changes. Although our baseline EEG complexity features demonstrated statistical stability, they might lack sufficient sensitivity to detect subtle and transient physiological changes induced by a single iTBS session. While EEG complexity measures, such as approximate entropy and multiscale entropy, effectively distinguish pathological states and predict clinical responses to multi-session rTMS [76], their utility for predicting immediate neurophysiological responses to single-session iTBS has not been systematically evaluated. Thus, it remains possible that they reflect broader, slower network dynamics relevant for clinical outcomes rather than transient, circuit-specific plasticity. Ultimately, interpreting single-session MEP or TEP shifts as definitive markers of rTMS-induced neuroplasticity may oversimplify or overlook the more complex and multiscale biological processes elicited by repeated stimulation.

Several limitations should be considered when interpreting our findings. First, although our dataset (73 sessions across 40 participants) is comparable or larger than recent ML studies predicting neurophysiological outcomes from TMS, it may still be insufficient for robustly training predictive models, partially explaining the wide confidence intervals observed in external validation. Second, our sample included only healthy controls, limiting direct generalizability to clinical populations, whose neurophysiological responses to iTBS may differ significantly. Finally, minor methodological differences between cohorts, such as the number of single-pulse TMS trials, post-TBS sampling windows, intervals between sessions, and slight variations in EEG preprocessing, may have introduced additional noise or cohort-specific variability. Addressing these issues with larger, clinically diverse samples, standardized protocols, and multimodal outcome measures will be critical to identify robust biomarkers of neurophysiological responses to rTMS protocol.

In summary, our findings demonstrate that while baseline rsEEG complexity measures can predict iTBS-induced changes in local cortical responses to a certain degree, the neurophysiological outcomes derived from single-session protocols are too unstable to support predictive models that generalize reliably beyond the training dataset. The decline in performance observed during external validation underscores the importance of having independent dataset and using a test-retest paradigm to avoid overly optimistic estimates of model accuracy. Overall, the considerable variability in individual responses remains a central barrier to optimizing the efficacy of rTMS. Addressing this challenge may require developing personalized stimulation strategies tailored to each individual’s unique brain anatomy and baseline neurophysiology, and systematically evaluating these strategies through multi-session protocols and richer, multimodal biomarkers that can effectively link robust behavioral improvements to their underlying neural mechanisms.

## Supplementary Material

This is a list of supplementary files associated with this preprint. Click to download.

- JNRSuppv01.docx

## Figures and Tables

**Table 1:** Performance of model selection and model validation of Cross-Cohort Experiments. Square brackets represent 95% confidence interval. Row a and b represent MEP Experiment. Row c and d represent LMFP Ratios Experiment. Row a and c represent model selection step whereas row b and d represent model

Model Name	Accuracy	Sensitivity	Specificity	F1	ROC AUC	PR AUC	Precision
MEP Cross-Cohort Experiment							
**a** LDA OA	0.72 [0.67, 0.76]	0.71 [0.63, 0.78]	0.72 [0.62, 0.82]	0.77 [0.72, 0.82]	0.71 [0.67, 0.76]	0.77 [0.73, 0.82]	0.84 [0.78, 0.90]
**b** LDA OA	0.65 [0.53, 0.77]	0.68 [0.48, 0.84]	0.60 [0.40, 0.80]	0.68 [0.51, 0.81]	0.65 [0.45, 0.80]	0.72 [0.59, 0.86]	0.68 [0.55, 0.85]
LMFP Ratios Cross-Cohort Experiment							
**c** Decision Tree	0.81 [0.76, 0.85]	0.82 [0.75, 0.89]	0.78 [0.69, 0.88]	0.83 [0.79, 0.88]	0.80 [0.76, 0.85]	0.79 [0.74, 0.84]	0.85 [0.79, 0.91]
**d** Decision Tree	0.69 [0.62, 0.79]	0.78 [0.70 0.87]	0.44 [0.22, 0.78]	0.79 [0.74, 0.86]	0.61 [0.49, 0.79]	0.79 [0.75, 0.88]	0.81 [0.75, 0.92]

## Data Availability

Data are available upon reasonable request.
